# Adhesions Following Pelvic Peritonitis

**Published:** 1888-07

**Authors:** J. M. Dutton

**Affiliations:** Boston, Mass.


J. M. Dutton, M. D.

Boston, Mass.

---

### ADHESIONS FOLLOWING PELVIC PERITONITIS.

Cures made accidentally or on general principles give very little pleasure to the practitioner who has once tasted the scientific satisfaction of exact symptom covering. To say that, confronted with such a disease, one gave a certain remedy which was followed by cure teaches nothing. It seems possible the report of such cases is an injury, since it encourages the make-shift habit which deals in generalities, when we are bound by the name we bear to treat symptoms alone. I report the following case, then, not for the value of its therapeutic work, but on account of the fresh proof it affords of the untrustworthy nature of diagnoses, founded upon supposed pathological conditions:

Mrs. H. E. L. (æt. fifty-six) had two years ago an acute attack of fever, with pain in the abdomen, spoken of by the attending physician as peritonitis. Since that time the patient had never been well, the most persistent and troublesome symptom being attacks of pain in the right inguinal region. The suffering was so violent that a pseudo-homœopathic doctor always gave Morphine. There were other outlying symptoms which I was able to control, but with their disappearance I had the mortification to find the attacks of pain increase in number and violence, my patient being forced to give up the care of her house, and with a nurse to assume the habits of a confirmed invalid.

Physical examination showed an adhesive band which contracted the vagina so much the posterior *cul-de-sac* could only be reached after some effort. The uterus was freely movable and in normal position. There was no evidence of a sensitive ovary on the right side, much less of the tumor the pseudo-homœopathic doctor had declared to be there. The conclusion seemed unavoidable, that the same inflammatory process which had formed the adhesive band in the vagina had thrown out plastic lymph in the region of the right broad ligament and ovary, which, contracting, had bound down these organs to the side of the pelvis. Motion of the limb dragging these parts still further out of place, the entangled nerves cried out, and from the nature of the case always would do so. With no hope of any permanent benefit, I abandoned a careful study of the symptoms as useless. Guided by the fact that Sulphur had relieved, temporarily, I gave Psorinum. To my surprise, the attacks of pain never appeared again. Now, after ten months, they have never returned. A better reward than my careless materialism deserved.

J. M. DUTTON, M. D.

